# Prevalence and associated factors of uncontrolled hyperlipidemia among Thai patients with diabetes and clinical atherosclerotic cardiovascular diseases: a cross-sectional study

**DOI:** 10.1186/s13104-021-05535-6

**Published:** 2021-03-25

**Authors:** Teeraboon Lertwanichwattana, Ram Rangsin, Boonsub Sakboonyarat

**Affiliations:** Department of Military and Community Medicine, Phramongkutklao College of Medicine, Bangkok, 10400 Thailand

**Keywords:** Uncontrolled hyperlipidemia, ASCVD, Diabetes, Prevalence, Risk factors

## Abstract

**Objectives:**

The research aimed to determine the prevalence and associated factors of uncontrolled hyperlipidemia among Thai patients with the disease and Clinical ASCVD.

**Results:**

A total of 1,527 Thai diabetic patients with a history of ASCVD were included in the study. Uncontrolled hyperlipidemia was detected among 1,216 patients (79.6%; 95% CI 77.6–81.7). The independent factors associated with uncontrolled hyperlipidemia included being female (adjusted odds ratio (AORs); 1.5, 95% CI 1.2–2.0), using thiazolidinedione (AORs; 1.7, 95% CI 1.1–2.7), community hospital (AORs; 4.3, 95% CI 1.0–18.0) and BMI level at 18.5–22.9 kg/m^2^ (AORs; 2.2, 95% CI 1.2–4.0), 23.0–24.9 kg/m^2^ (AORs; 1.8 95% CI 0.9–3.3), 25.0–29.9 kg/m^2^ (AORs; 2.3 95% CI 1.3–4.3) and ≥ 30 kg/m^2^ (AORs; 2.5 95% CI 1.3–4.9).

## Introduction

Dyslipidemia (DLP) is one of the major risk factors of Clinical Atherosclerotic Cardiovascular Diseases (ASCVD) which is a group of diseases with one of the major mortality incidences in the world [1]. The World Health Organization (WHO) reported that cardiovascular diseases were the most common cause of death globally; however, these diseases could be attenuated by lifestyle modification and medication used [2, 3]. DLP is defined by the elevated levels of plasma cholesterol, triglycerides, or both, or reduced levels of high density lipoprotein cholesterol (HDL-C) [4]. Therefore, DLP can contribute to atherosclerosis [5, 6]. Then these vascular consequences proceed to Clinical ASCVD comprising ischemic heart disease (IHD), ischemic stroke, transient ischemic attack (TIA) and peripheral artery disease (PAD) [5, 6]. Diabetes mellitus (DM) serves an important role in DLP by decreasing catabolism of triglyceride rich lipoproteins of intestinal and hepatic origin [7]. Therefore, DM affects lipid metabolism increasing cholesterol levels especially among patients with DM and DLP [7]. However, limited information is available regarding the prevalence of uncontrolled DLP among patients with DM with ASCVD, especially in Thailand. We aimed to determine the prevalence and associated factors of uncontrolled hyperlipidemia among Thai diabetic patients with Clinical ASCVD.

## Main text

### Methods

The data were retrieved from the database: an Assessment on Quality of Care among Patients Diagnosed with Type 2 Diabetes (T2D) and Hypertension Visiting Hospitals under the Authority of the Ministry of Public Health (MoPH) and Hospitals in Bangkok, Thailand, (the Thailand DM/HT study) after obtaining permission from the National Health Security Office (NHSO) and the Medical Research Network of the Consortium of Thai Medical Schools (MedResNet). A standardized case report form was used to obtain the required information from medical records of DM treatment between February and May in 2018 and was sent to the Thailand DM/HT study of the NHSO Central Data Management Unit in Nonthaburi. This study was reviewed and approved by the Royal Thai Army Medical Department Institutional Review Board, reference number R045h/63_Exp.

The inclusion criteria comprised patients with T2D aged at least 18 years with history of Clinical ASCVD including IHD, ischemic stroke, TIA and PAD. Any patient who had participated in a clinical trial was excluded. Those patients may have received trial medication or placebo, influencing the outcome of the study. The participant population totaled 1,527 patients. Data collected included demographics (sex, age, occupation, religion and health scheme), weight, height, body mass index (BMI), smoking behavior, alcohol consumption behavior, hospital level, diabetic complications such as diabetic retinopathy, diabetic nephropathy, diabetic neuropathy, blood chemistry data including fasting plasma blood glucose, hemoglobin A1c (HbA1c), lipid profile including total cholesterol, triglyceride (TG), HDL-C and low density lipoprotein cholesterol (LDL), history of anti-hyperglycemic, anti-hypertensive and lipid-lowering drug use and glomerular filtration rate calculated using the epidemiology collaboration formula.

The study included those patients with a diagnosis of T2D and history of Clinical ASCVD. All patients needed to receive ongoing medical treatment in a registered hospital. Thai hospitals use the American Diabetes Association standard to diagnose and treat patients with T2D [8]. Criteria to diagnose diabetes were defined as one of the following indicators. The first was fasting plasma glucose ≥ 126 mg/dL (7.0 mmol/L). Fasting is defined as no caloric intake for at least 8 h. The second was 2-h postprandial glucose (2-h PG) ≥ 200 mg/dL (11.1 mmol/L) during the oral glucose tolerance test which uses a glucose load containing the equivalent of 75 g anhydrous glucose dissolved in water. In a patient with classic symptoms of hyperglycemia or hyperglycemic crisis, a random plasma glucose comprises ≥ 200 mg/dL (11.1 mmol/L). The first three tests are confirmed by repeat testing at a second visit. Clinical ASCVD is defined by one of the following: (1) IHD including ST-elevation myocardia infraction, nonST-elevation myocardial infraction and unstable angina, (2) ischemic stroke and TIA and (3) PAD [9].

Extrapolating from the available data, an absolute reduction to an LDL-C level, 1.8 mmol/L (less than 70 mg/dL) or at least a 50% relative reduction in LDL-C provides the best benefit in terms of CVD reduction [10]. Therefore, the target LDL-C of patients with very high cardiovascular risk as participants in the study had less than 70 mg/dL or a ≥ 50% reduction from baseline LDL-C. Thus, uncontrolled hyperlipidemia among patients with T2D and Clinical ASCVD was defined by LDL-C level ≥ 70 mg/dL (1.8 mmol/L) [10].

Data were analyzed using IBM SPSS Statistics for Windows, Version 22.0. Categorical data were presented as number and percentage. Prevalence was analyzed using descriptive statistics and reported as percentage and 95% confidence interval (CI). Multivariate analysis was performed using logistic regression analysis, and a *p*-value less than 0.05 was considered statistically significant.

### Results

A total of 38,568 patients with T2D were included in the Thailand DM/HT study in 2018. Of these, 1,527 patients with T2D with a history of Clinical ASCVD were enrolled in the present study. The patients’ baseline characteristics are presented in Table 1. Among enrolled patients, average age was 66.6 ± 10.3 years, and 842 (55.1%) patients were female. Most participants with hyperlipidemia were treated by statin and fibrates accounting for 80%. Of the 1,527 eligible participants, 1,216 patients did not achieve the LDL-C level goal according to 2018 AHA/ACC guidelines. Overall prevalence of uncontrolled hyperlipidemia (LDL-C ≥ 70 mg/dL) in the study was 79.6% (95% CI 77.6–81.7). This prevalence increased with older age and was more common among females. After adjusting for potential confounders, risk factors associated with uncontrolled hyperlipidemia were being female (adjusted odds ratio (AORs); 1.5, 95% CI 1.2–2.0), using thiazolidinedione (AORs; 1.7, 95% CI 1.1–2.7), community hospital (AORs; 4.3, 95% CI 1.1–18.0) and higher BMI levels (Tables 2 and 3).

**Table 1:** Demographic characteristics of participants

Characteristics	n (%)
Gender	
Male	685 (44.9)
Female	842 (55.1)
Age (years) (min–max)	(33.0–96.0)
< 60	383 (25.1)
60–69	537 (35.2)
70–79	431 (28.2)
≥ 80	176 (11.5)
Age (years) (mean ± S.D.)	66.6 ± 10.3
Hospital level	
Standard/Advanced hospital	1473 (96.5)
Community hospital	54 (3.5)
Scheme	
Universal healthcare coverage	1193 (78.1)
Civil servant medical benefit	294 (19.3)
Social security	33 (2.2)
Others	7 (0.5)
Regions	
North	369 (24.2)
Central	578 (37.8)
Northeast	306 (20.0)
South	275 (18.0)
Occupation	
Unemployed	660 (44.7)
Indoor occupation	167 (11.3)
Outdoor occupation	649 (44.0)
BMI group (kg/m^2^) (mean ± SD)	25.6 ± 4.6
< 18.5	385 (25.9)
18.5–22.9	64 (4.3)
23.0–24.9	294 (19.8)
25.0–29.9	505 (33.9)
≥ 30.0	240 (16.1)
Smoking status	
Current smoker	64 (4.3)
Ex-smoker	241 (16.1)
Never	1189 (79.6)
Alcohol drinking	
Current drinkers	55 (3.7)
Ex-drinker	170 (11.5)
Never	1249 (84.7)
Hypertension	
No	148 (9.7)
Yes	1379 (90.3)
Gout	
No	1382 (90.5)
Yes	145 (9.5)
Diabetic kidney disease	
No	1334 (87.4)
Yes	193 (12.6)
Diabetic retinopathy	
No	1390 (91.0)
Yes	137 (9.0)
Stroke	
No	1069 (67.8)
Yes	492 (32.2)
Ischemic heart diseases (IHD)	
No	552 (36.1)
Yes	975 (63.9)
Peripheral arterial diseases (PAD)	
No	1400 (91.7)
Yes	127 (8.3)
Number of clinical ASCVD	
1	1460 (95.6)
≥ 2	67 (4.4)

Clinical ASCVD: Clinical Atherosclerotic Cardiovascular Diseases including Stroke, IHD, PAD

*BMI* body mass index, *SD* standard deviation, *min* minimum, *max* maximum, *kg/m*^*2*^ kilogram per meter squared

**Table 2:** Univariate analysis for factor associated with uncontrolled hyperlipidemia among T2D patients with clinical ASCVD

Factors	LDL-C level (mg/dL)		Crude Odds Ratio	95% CI	*p*-value
	≥ 70	< 70			
	n (%)	n (%)			
Gender					
Male	521 (76.1)	164 (23.9)	1.0		
Female	695 (82.5)	147 (17.5)	1.5	1.2–1.9	0.002
Age (years) (mean ± S.D.)	66.5 ± 10.3	67.1 ± 10.3	1.0	1.0–1.0	0.392
< 60	311 (81.2)	72 (18.8)	1.0		
60–69	427 (79.5)	110 (20.5)	0.9	0.6–1.3	0.527
70–79	335 (77.7)	96 (22.3)	0.8	0.6–1.1	0.222
≥ 80	143 (81.3)	33 (18.8)	1.0	0.6–1.6	0.989
Scheme					
Universal healthcare coverage	960 (80.5)	233 (19.5)	1.0		
Civil servant medical benefit	225 (76.5)	69 (23.5)	0.8	0.6–1.1	0.133
Social security	24 (72.7)	9 (27.3)	0.6	0.3–1.4	0.274
Hospital level					
Standard/advanced hospital	1168 (79.3)	305 (20.7)	1.0		
Community hospital	48 (88.9)	6 (11.1)	2.1	0.9–4.9	0.092
Regions					
North	289 (78.3)	80 (21.7)	1.0		
Central	461 (79.8)	117 (20.2)	1.1	0.8–1.5	0.595
Northeast	242 (79.3)	242 (29.7)	1.1	0.7–1.5	0.746
South	224 (81.5)	224 (18.5)	1.2	0.8–1.8	0.329
Occupation					
Unemployed	519 (78.6)	141 (21.4)	1.0		
Indoor occupation	138 (82.6)	29 (17.4)	1.3	0.8–2.0	0.254
Outdoor occupation	517 (79.7)	132 (20.3)	1.1	0.8–1.4	0.648
BMI (kg/m^2^) (mean ± SD)	25.7 ± 4.6	25 ± 4.7	1.0	1.0–1.1	0.016
< 18.5	42 (65.6)	22 (34.4)	1.0		
18.5–22.9	310 (80.5)	75 (19.5)	2.2	1.2–3.8	0.008
23.0–24.9	222 (75.5)	72 (24.5)	1.6	0.9–2.9	0.105
25.0–29.9	412 (81.6)	93 (18.4)	2.3	1.3–4.1	0.003
≥ 30.0	201 (83.8)	39 (16.3)	2.7	1.5–5.0	0.002
Smoking status					
Never	953 (80.2)	236 (19.8)	1.0		
Current smoker	47 (73.4)	17 (26.6)	0.7	0.4–1.2	0.195
Ex-smoker	189 (78.4)	52 (21.6)	0.9	0.6–1.3	0.542
Alcohol drinking					
Never	1016 (81.3)	233 (18.7)	1.0		
Current drinker	39 (70.9)	16 (29.1)	0.6	0.3–1.0	0.057
Ex-drinker	121 (71.2)	49 (28.8)	0.6	0.4–0.8	0.002
Hypertension					
No	115 (77.7)	33 (22.3)	1.0		
Yes	1101 (79.8)	278 (20.2)	1.1	0.8–1.7	0.540
Gout					
No	1106 (80.0)	276 (20.0)	1.0		
Yes	110 (75.9)	35 (24.1)	0.8	0.5–1.2	0.237
Diabetic kidney disease					
No	1057 (79.2)	277 (20.8)	1.0		
Yes	159 (82.4)	34 (17.6)	1.2	0.8–1.8	0.311
Diabetic retinopathy					
No	1105 (79.5)	285 (20.5)	1.0		
Yes	111 (81.0)	26 (19.0)	1.1	0.7–1.7	0.672
Biguanides					
No	411 (81.4)	94 (18.6)	1.0		
Yes	805 (78.8)	217 (21.2)	0.8	0.6–1.1	0.232
Sulfonylurea					
No	581 (78.6)	158 (21.4)	1.0		
Yes	635 (80.6)	153 (19.4)	1.1	0.9–1.4	0.341
Thiazolidinedione					
No	1061 (78.8)	286 (21.2)	1.0		
Yes	155 (86.1)	25 (13.9)	1.7	1.1–2.6	0.023
Alpha—glucosidase Inhibitor					
No	1207 (79.6)	309 (20.4)	1.0		
Yes	9 (81.8)	2 (18.2)	1.2	0.2–5.4	0.857
DPP—4 Inhibitor					
No	1194 (79.8)	302 (20.2)	1.0		
Yes	22 (71.0)	9 (29.0)	0.6	0.3–1.4	0.230
SGLT2 inhibitor					
No	1215 (79.7)	310 (20.3)	1.0		
Yes	1 (50.0)	1 (50.0)	0.3	0.1–4.1	0.335
Insulin					
No	905 (79.8)	229 (20.2)	1.0		
Yes	311 (79.1)	82 (20.9)	1.0	0.7–1.3	0.776
Statin					
No	198 (81.8)	44 (18.2)	1.0		
Yes	1018 (79.2)	267 (20.8)	0.8	0.5–1.2	0.358
Fibrates					
No	1153 (79.6)	296 (20.4)	1.0		
Yes	63 (80.8)	15 (19.2)	1.1	0.6–1.9	0.798
FPG (mg/dL)	148.5 ± 55.9	146.9 ± 51.9	1.0	1.0–1.0	0.655
HbA1c (%) (mean ± SD)	7.8 ± 2.0	7.7 ± 1.9	1.0	1.0–1.1	0.331
< 7.0	441 (77.5)	128 (22.5)	1.0		
7.0–7.9	266 (79.4)	69 (20.6)	1.1	0.8–1.6	0.504
8.0–8.9	191 (83.4)	38 (16.6)	1.5	1.0–2.2	0.064
9.0–9.9	107 (80.5)	26 (19.5)	1.2	0.7–1.9	0.460
≥ 10.0	141 (77.9)	40 (22.1)	1.0	0.7–1.5	0.911
Number of ASCVD					
1	1162 (79.6)	298 (20.4)	1.0		
≥ 2	54 (80.6)	13 (19.4)	1.1	0.6–2.0	0.841

*LDL-C* low-density lipoproteins cholesterol, *SD* standard deviation, *Clinical ASCVD* clinical atherosclerotic cardiovascular diseases, *BMI* body mass index, *kg/m*^*2*^ kilogram per meter squared, *mg/dL* milligrams per deciliter

**Table 3:** Multivariate analysis for factor associated with uncontrolled hyperlipidemia among T2D patients with clinical ASCVD

Factors	LDL-C level (mg/dL)		Adjusted Odds Ratio	95% CI	*p*-value
	≥ 70	< 70			
	n (%)	n (%)			
Gender					
Male	521 (76.1)	164 (23.9)	1.0		
Female	695 (82.5)	147 (17.5)	1.5	1.2–2.0	0.002
Hospital level					
Standard/advanced hospital	1168 (79.3)	305 (20.7)	1.0		
Community hospital	48 (88.9)	6 (11.1)	4.3	1.0–18.0	0.048
Thiazolidinedione					
No	1061 (78.8)	286 (21.2)	1.0		
Yes	155 (86.1)	25 (13.9)	1.7	1.1–2.7	0.024
BMI (kg/m^2^)					
< 18.5	42 (65.6)	22 (34.4)	1.0		
18.5–22.9	310 (80.5)	75 (19.5)	2.2	1.2–4.0	0.016
23.0–24.9	222 (75.5)	72 (24.5)	1.8	0.9–3.3	0.074
25.0–29.9	412 (81.6)	93 (18.4)	2.3	1.3–4.3	0.007
≥ 30.0	201 (83.8)	39 (16.3)	2.5	1.3–4.9	0.007

*LDL-C* low-density lipoproteins cholesterol, *Clinical ASCVD* clinical atherosclerotic cardiovascular diseases *BMI* body mass index, *kg/m*^*2*^ kilogram per meter squared, *mg/dL* milligrams per deciliter

### Discussion

These results demonstrated important implications to the Thai public health system because uncontrolled hyperlipidemia is a major risk factor for recurrent ASCVD and its complications. Our study revealed that the essential evidence of a high prevalence of uncontrolled hyperlipidemia among Thai patients with T2D and Clinical ASCVD was approximately 80%. Additionally, we found that being female, using TZD, hospital level and higher BMI level were associated with uncontrolled hyperlipidemia.

The present study showed that four fifths of the study participants could not control their LDL-C. This situation could be explained by several points. Firstly, this study was conducted among patients receiving a diagnosis of diabetes. In the setting of insulin resistance and DM, lipoprotein lipase activity, a rate-limiting enzyme in the hydrolysis of triglyceride, is reduced [11]. Very low-density lipids (VLDL) can be incompletely lipolyzed, yielding increased serum levels of VLDL remnants, after catabolism and triglyceride exchange were completed. The LDL particles become progressively more enriched with triglycerides, which are lipolyzed to smaller, denser and more numerous particles. These smaller particles are more atherogenic than those of larger size and less dense LDL particles [12]. Related studies in five Asian countries reported that the prevalence of uncontrolled LDL-C among patients with a history of coronary heart diseases was 92.0%; however, after 12 months follow-up, 33.8% of patients reached LDL-C goals (< 100 mg/dL) [13]. Compared with a pool analysis of observational studies, the present study reported a lower prevalence of uncontrolled hyperlipidemia [13]. One related study in China found a lower prevalence of uncontrolled hyperlipidemia (63.8%) among those experiencing acute coronary syndrome undergoing statin treatment (> 2 weeks) [14]. Additionally, compared with one related study in the US, the prevalence of uncontrolled hyperlipidemia in the study was comparable with those in the US study accounting for 74.7%. However, uncontrolled hyperlipidemia in the US study was defined by an LDL-C level > 100 mg/dL because the participants were only patients with DM without history of ASCVD [15].

The present study showed that patients with uncontrolled hyperlipidemia tended to be higher among females. Similar to the US study, sex differences in the prevalence of, and trends in cardiovascular risk factors, treatment, and control in the US, 2001 to 2016, indicated men were more likely to be treated and to have controlled DLP, especially at older age [16]. The phenomenon can be explained by several ways. Firstly, several epidemiologic studies found that postmenopausal women had different lipid profiles when compared with premenopausal women [17–19]. In the study, the average age of women with DM and history of ASCVD was 67.3 ± 10.6 years and menopausal. The reason for uncontrolled hyperlipidemia among females was the menopausal transition [20, 21]. Additionally, the causes of uncontrolled hyperlipidemia are mostly found among females experienced with Clinical ASCVD because females with DM have a cardiovascular mortality risk greater than males with DM: 20.9 versus 14.9% [22–24]. Moreover, postmenopausal women lack estrogen having two effects on lipid metabolism. Firstly, estrogen depends on regulation of LDL receptors resulting in increased LDL particle clearance by hepatocytes; thus, decreasing plasma LDL-C [25]. Another study revealed that estrogen receptors are present in adipocytes which have a 17-beta-estradiol [26]. The high level of this hormone directly affects marked inhibition of adipose tissue lipoprotein lipase (LPL) activity and hormone-sensitive lipase (HSL) [27]. LPL initiates chylomicron metabolism and conversion of VLDL to LDL [28]. HSL was studied in mice showing that HSL had compensatory mechanisms to increase LDL receptor expression [29]. Thus, elderly females tended to have high LDL levels compared with males.

Another factor associated with uncontrolled hyperlipidemia among patients with DM and Clinical ASCVD was thiazolidinediones (TZDs) use. TZDs have the potential to benefit the full “insulin resistance syndrome” associated with DM [30]. According to Thai rational drugs use in 2018, pioglitazone hydrochloride was the only drug in the TZD group. Some studies have revealed that treatment with pioglitazone modestly increased LDL-cholesterol levels by ~ 10 to 15% and increased LDL particle size [31, 32]. Another study in Germany, reporting that pioglitazone reduced atherogenic dense LDL particles among patients with T2D, showed that pioglitazone decreased the amount of LDL-6 (the densest LDL subfraction), resulting in larger and less dense LDL particles [33]. However, the present study found that the TZDs were administered among patients with DM and history of ASCVD, who had uncontrolled hyperlipidemia. The reason may be from potential benefits on the secondary complications of DM. Another study found that among patients with T2D, pioglitazone plus sulfonylurea significantly improved HbA1C and fasting plasma glucose levels with beneficial effects on serum triglyceride and HDL-C levels [34].

Hospital levels in Thailand are classified in two categories: standard/advanced level and community level. We found that community level hospitals were significantly associated with uncontrolled hyperlipidemia among patients with DM and history of Clinical ASCVD. This finding may reflect the fact that community level hospitals are located in district areas and have limited resources including specialists, various types of medication and laboratory testing [35]. Patients recovering from diseases were referred to and received regular medication in community hospitals [4]. At the community level, patients’ cholesterol levels may be under monitored. In addition, a related survey conducted in a primary care setting in Thailand showed that necessary and routine aspects of diabetic care were not performed by the healthcare systems regularly [36]. Thus, a higher prevalence of uncontrolled hyperlipidemia was observed among community hospitals when compared with the standard/advanced hospitals.

Finally, we found a dose response relationship between higher BMI level and uncontrolled hyperlipidemia. This result was similar to that of related studies in Spain and Indonesia showing that BMI was associated with a high LDL level [37, 38]. Another study in Thailand indicated the prevalence of small dense LDL increased obesity status in a Thai population which may be assumed to be an increased BMI [39]. The phenomenon can be explained by metabolic processes. Hyperlipidemia enhancing in obesity involves elevated fasting and postprandial TG combined with the essential small dense LDL and low HDL-C. An increase in TG level may be the principal cause of other lipid abnormalities because it leads to delayed clearance of the TG-rich lipoproteins and formation of small dense LDL [40–42]. However, some studies have reported that LDL-C showed no significant correlation with BMI level [43, 44].

The strength of this study included the scope for uncontrolled hyperlipidemia among patients with DM and history of ASCVD. One implication of the study is to reduce the prevalence of uncontrolled hyperlipidemia, resulting from improving DM management. Moreover, the health literacy of our patients should be improved to increase awareness of their behaviors especially body weight control. Eventually, healthcare services access by patients with DM especially at community levels should be adjusted and improved to alleviate their cardiovascular complications.

## Limitations

This study employed a cross-sectional design, so the results could show only factors associated with uncontrolled hyperlipidemia. We were aware of missing data from this observational study. However, some data were missing as from the nationwide observational study, so few associations between factors and outcomes could be presented.

## Data Availability

The datasets generated and/or analyzed during the current study are available at http://www.damus.in.th after the permission of the Thailand DM/HT study of the MedResNet.

## Abbreviations

DM: Diabetes mellitus
HT: Hypertension
ASCVD: Atherosclerotic cardiovascular diseases
SBP: Systolic blood pressure
DBP: Diastolic blood pressure
CI: Confidence interval
BMI: Body mass index
MoPH: Ministry of Public Health
NHSO: National Health Security Office
SD: Standard deviation
LDL: Low density lipoprotein cholesterol
HbA1c: Hemoglobin A1c
